# Medical Opinion and Movement

**Published:** 1909-08-14

**Authors:** 


### Medical Opinion and Movement.

Dr. Th. Goldenberg has conceived a novel method of treating Tuberculous Abscesses by means of setting free the proteolytic ferments contained in the leucocytes. His results are published in the *Münchner Medizinsche Wochenschrift.* According to Heile, the good results obtained by injections of iodoform emulsion in glycerine are due to the leucocytosis which they excite. The leucocytes liberating proteolytic ferments into the abscess cavity assist the absorption of the pus, and in this way the cavity is emptied and becomes lined with granulations. Otherwise the tuberculous abscess is poor in leucocytes and in ferments. Acting on this supposition, Jochmann and Bätzner have treated such abscesses by injecting ferments directly into them. Goldenberg has, however, adopted the following means, and claims to have obtained very satisfactory results. He injects into the cavity nucleinate of soda, which excites a considerable local leucocytosis. He then treats the abscess cavity with the *x*-rays, by which the white corpuscles are destroyed and the ferments liberated within the abscess cavity. Instead of nucleinate of soda he has also used as a chemiotactic a combination of silver salts and albumen with equally satisfactory results.

---

There are many Morbid Conditions in which the keynote is the absence of equilibrium between the food absorbed and the energy expended by the organism. If the quantity of food absorbed is insufficient a condition of marasmus results, whereas an excess leads to obesity or to one of a group of affections characterised by an inert and insufficient metabolism. In the treatment of these last-mentioned affections an endeavour is usually made, either directly or indirectly, to restrict the diet, but rarely to cause an increase in the consumption of energy. In many cases the patients are in a depressed and weakened condition, and are unable to make the psychomotor effort necessary in order to increase their expenditure of energy by muscular work. Reasoning on these lines, Dr. J. Bergonié has attempted to treat such cases, especially rheumatic, gouty, and glycosuric, by causing muscular contractions by means of the electric current. He has given an account of his work at a recent meeting of the Académie des Sciences. He uses an alternating current of 8 to 12 volts, with a frequency of 40 to 100 per second and a strength of 50 milliamperes. He applies the current by means of very large electrodes covering a large portion of the body. By these rhythmic contractions are induced in all the chief muscular portions of the body—arms, thighs, legs, back, and shoulders. They are sufficiently powerful to raise the body, even when weighted at the thighs with 40 kilos. or more, and at the same time they are said to be quite painless. The respiratory exchanges are enormously increased. With the diet remaining the same as was sufficient for equilibrium before treatment, the loss in fatty tissue is rapid, and at the same time the strength and power of resistance to fatigue increase.

An interesting case of persistent and severe Gastric Crises of tabetic origin cured by section of the posterior roots of the seventh to the tenth dorsal nerves is recorded by Drs. Förster and Küttner in the *Beitrage z. Klinischen Chirurgie.* The case was that of a man aged 47, suffering from tabes dorsalis. In addition to the usual symptoms he developed gastric crises, which became so frequent and lasted so long that the patient did not have more than five or six days' respite from them in the course of a month, and that thanks to a dose of 72 centigrammes of morphia. The crises were accompanied by abundant vomiting to the extent of 1½ litres of mucus, gastric juice, and bile. The authors, in discussing the nature of these gastric crises, point out that they are essentially sensory in origin, the motor manifestations—vomiting, hyper-secretion of mucus and gastric juice, and exaggerated abdominal reflexes being determined in a reflex manner. The sensory nerves of the stomach pass partly by the vagus and partly by the sympathetic—that is by the cardiac plexus, the great splanchnic, and the rami communicantes to the seventh, eighth, and ninth dorsal roots. The cutaneous hyperæsthesia and reflex hyperexcitability of the abdominal muscles accompanying the crises also pointed to these roots as the source of irritation. As the patient had been brought into a state of pronounced cachexia, and his condition was considered critical, he agreed to the operation of division of these posterior dorsal roots. The operation was performed in two stages. At the first operation a laminectomy of the fifth to the tenth dorsal vertebræ was carried out, and bearings taken for the subsequent division of the nerve-roots. This operation was followed by such an exacerbation of the vomiting that the second was undertaken on the eleventh day. The success was immediate and astounding. The pains, nausea, and disgust for food disappeared entirely, and the appetite returned. The patient rapidly put on weight, and continues still to do so, three months after the operation. There persists still some slight intestinal colic, and there is, of course, an area of complete cutaneous anæsthesia from a little below the xiphoid to the umbilicus.

---

With a view to determine the therapeutic properties of Cerium Oxalate, especially in cases of vomiting, Dr. G. Baehr and H. Wessler have carried out a series of experiments with this drug. As cerium oxalate of commerce contains also oxalates of lanthane, thorium, and other compounds, they first examined the drug from the point of view of its toxicity. They find that all these oxalates are quite harmless for dogs, and that the cerium oxalate itself can be given in large doses, as much as 50 grammes, without producing any abnormal symptoms, except an increase in the daily quantity of fæces, which, they remark, may result from the ingestion of any inert powder, if given in sufficiently large doses. In regard to the anti-emetic effect of the drug, they find that it is unable to arrest the vomiting consequent on the hypodermic injection of apomorphine—that is to say, vomiting of central origin. But, on the other hand, it is quite effectual against vomiting due to a local irritation of the gastric mucous membrane, such as results from the administration of ipecacuanha. The drug, to be efficacious, must, however, be given in large doses, so as to form a sort of covering to the wall of the stomach. These experiments, therefore, controvert the idea that cerium oxalate acts as a sort of sedative to the nervous system, such as would appear to be indicated in cases of vomiting in pregnancy, and this supposition is further negatived by the fact that the drug is almost entirely insoluble in the biological fluids, and can therefore only be absorbed in very small quantities. The action of cerium oxalate appears to be purely local and mechanical. The authors show a close analogy in these respects between cerium oxalate and bismuth subnitrate, and think that the former might be used with advantage in such cases in which the subnitrate of bismuth finds more usual employment —that is, in cases of alcoholic gastritis and gastric ulcer—but it must be given in the same doses to be at all effective.

---

THE dangers which may result from prolonged exposure to currents of high frequency and high tension, such as are necessary in the working of wireless telegraphy, are touched upon by Bellier in the *Archives de Médecine Navale*. Men who constantly manipulate the apparatus for the emission of the Hertzian waves are frequently attacked by visual disturbances with more or less severe conjunctivitis, seemingly the direct result of the electric sparks. At times the conjunctivitis is complicated by keratitis and a resultant corneal opacity. Sometimes, in addition, exposure to the electric flashes causes eczema of the eyelids and wrists. Palpitations of nervous origin have also been recorded. Mayhap further experience will show that these complications do not exhaust the catalogue of diseases to which wireless telegraphy can give rise, but at present the ocular phenomena would seem definitely established. With the object of preserving the eyes from the deleterious action of the chemical and ultra-violet rays, the author is of opinion that blue or preferably yellow-coloured glasses should be served out to all manipulators of these hurtful currents.

---

DEMELIN, in *le Nord Médical*, publishes an interesting article on the practical Diagnosis of the Engagement of the Fœtus. After calling attention to the neglectful manner in which this subject is treated in most text-books, which rarely define the condition, he proceeds to give his own definition. By the " engagement " he understands the descent of the presenting part from the entry of the true pelvis or superior peloic strait, to the pelvic floor. Engagement and descent are thus, in his estimation, synonymous terms. In practice it is necessary to be able to recognise when engagement has taken place and the extent to which it has progressed, and the author calls attention to the ways in which these can be estimated. If examination be carelessly carried out a caput succedaneum descending low down may be mistaken for the presenting part, which may be still lying above the superior pelvic strait. There can be no engagement if the presenting part is mobile, and can be felt at or above the superior strait, as also if the pelvis be empty and there be any difficulty in feeling the presenting part, per vaginam. A useful help in estimating the degree of the engagement is afforded by the " Shoulder-sign," described by Fabre. If a frozen full-term fœtus be cut sagitally, so as to bisect the biparietal and bisacromial diameters, the distances between the point where these diameters are cut and the suboccipital point will be found to be two and five inches respectively. If, therefore, during labour the anterior shoulder of the fœtus be recognised five inches above the symphysis pubis, the lowest part of the head lies at the superior strait. If the shoulder is found three inches above the symphysis the biparietal diameter must lie in the plane of the superior strait. If the shoulder is placed between three and five inches above the joint only a part of the space which separates the suboccipital point from the centre of the biparietal diameter is situated below the superior strait. No doubt these measurements cannot be rigidly applied in all cases, but after allowing for errors due to asynclitism and lateral deviations of the head, they will be found very useful in practice, especially if corroboratory evidence be obtained by a vaginal examination, carried out according to the rules laid down by the author.

---

IT is reported from the Surgical Society of Paris that a Myxelial Fungus, the *Hemispora stellata* first described by Villemin, may produce lesions that resemble tuberculosis histologically. So apt is one to put down giant-cells as probably tuberculous in origin that one is apt to forget that other conditions than tubercle can produce giant-celled systems also. M. Auvray showed a case in point, having investigated it with great thoroughness to prevent fallacy. The patient was a man suffering from what seemed to be an inflammatory swelling in the neck, beneath the angle of the jaw. It was under the skin, but near the surface, and it had persisted so long that a diagnosis of tuberculous lymphatic gland appeared to be fairly probable. The mass was finally excised and examined microscopically, in addition to which cultures were made from it too. Histologically it could very easily have been mistaken for a tuberculous tissue, presenting well-marked giant cells, surrounded by epithelial cells. The cultivations however yielded no trace of tubercle bacilli, but gave pure growths of *Hemispora stellata*. This is not the first case of the kind reported. It would seem, therefore, that it would be wise, in making a diagnosis of tuberculosis of lymphathic glands, and other tissues, not to rely solely upon the presence of giant-celled systems, but to go a step further and stain sections containing such giant cells for tubercle bacilli which would be brought out by the well-known Ziehl-Neelsen method.

A NEW method of treatment for hydrocele, similar to the old one by injection of iodine, and with which the author claims to have obtained excellent results, is described by Marcozzi in *les Annales des Maladies des Organes Génito-urinaires.* This consists in introducing small fragments of magnesium into the tunica vaginalis after tapping the hydrocele, the irritant action and slow absorption of the metal effecting a cure. The magnesium is first washed in ether and then boiled in distilled water. After puncturing the sac, the cavity is washed out with warm water, and a small quantity of fluid left behind to prevent the walls of the sac from coming into contact with each other. The fragments of the metal, to the extent of a drachm in weight, are then introduced into the sac through the cannula. The water is next removed and the scrotum suspended. At the end of about fourteen days the magnesium will start being absorbed. A cure usually results without pain, swelling, inflammation, or even a rise of temperature.

---

BAR in the *Concours Médicale* publishes a study of facial paralysis in the new-born. The most frequent cause of the condition is the asymmetrical application of the forceps, but it may result, though this is much less common, from version. Facial paralysis is rarely seen when the blades of the forceps are applied symmetrically, but in difficult labour when one blade grips the forehead and the other lies behind the ear, two lesions are produced and followed frequently by facial palsy. The lesion which causes this complication is in almost all cases that behind the ear where the nerve is crushed at its exit from the stylo-mastoid foramen. Were the frontal lesion the causal agent, convulsions and hemiplegia would almost certainly occur, which is not the case. The paralysis is therefore of peripheral origin, and is one of three kinds, superior, inferior and total, according to whether the superior, inferior, or both peripheral branches are implicated. The inferior branch is most commonly affected because it is the most superficial and injured by slight compression. A deviation of the labial commissure is the result. It is more frequent to meet with injury of both nerves than with that of the superior branch alone. This last is the most grave, but owing to this nerve lying deeper than the inferior branch it will be cured the earlier when both are affected. The presence of a superior palsy is shown by the child keeping one of its eyes open. All three forms of paralysis are due to contusion and compression, and not to section of the nerve. They are therefore transient, and pass off in a few days. In this they differ from the paralysis of the brachial plexus which sometimes follows version, and is due to actual tearing of the nerves as the result of traction on the arm. Since it passes off spontaneously, treatment of facial palsy is usually not needed. The eye must, however, be attended to when the superior branch is affected, and it may at times be necessary to stimulate a paralysis which is passing away very slowly with applications of gentle faradism. The author ends by calling attention to the advantage of the use of forceps over version in difficult cases, seeing that the paralysis which follows the former is always transient, whereas traction on the arm may lead to permanent injury.

---

AN interesting case was recently shown at a meeting of the Sociéte de Chirurgie by Legrain, in which the operation of Trephining had been performed on eleven different occasions for various phenomena which resulted from a fall on the head. The first operation was undertaken for the relief of fits of absent-mindedness which sometimes lasted several hours. Other operations followed in due course, when further symptoms of vertigo, and, later, Jacksonian epilepsy, ensued. At first the patient's condition appeared to be improved by the operations, but during the past ten years he has suffered from convulsive seizures and permanent contractures. At the present time the cerebral pulse can be seen beating under the skin covering the opening in the skull, which extends from the left parietal region to the parieto-occipital suture. On coughing, a large cerebral hernia can be seen to form. The patient shows no mental symptoms, but is affected by an incomplete right-sided hemiplegia with permanent contractions, but no changes in sensibility. It is uncertain whether he suffered from epilepsy prior to the accident or not, so that it is impossible to be certain that the Jacksonian epilepsy was the result of trauma, whether from the accident or from the operative interference. The consensus of opinion was that further operative treatment would be of no avail, and it is possible that the patient also was content to do without his twelfth experience of trephining.

---

AT a recent meeting of the Paris Surgical Society, Chavannaz of Bordeaux showed an interesting case of Intra-peritoneal Myxoma, in a patient of 35 years of age. The patient, who, when first seen, showed a large, ill-defined mass in the right iliac fossa, had for several years past complained of vague abdominal pains, on occasions simulating an attack of sub-acute appendicitis. These occasional attacks, or crises as the author calls them, were attended with obstinate constipation, and an examination of the fæces showed small masses of hard dry material almost resembling phosphatic concretions. During the attacks there was no fever. When seen by the surgeon, the patient presented an emaciated, sallow appearance, and a diagnosis of sub-acute appendicitis with adhesions was made. At the operation the mass was found to consist of a much hypertrophied appendix at the base of which lay a fairly large myxomatous nodule. The author thinks that the symptoms were due to the appendix primarily, and traces the source of the trouble to a catarrhal appendicitis. The mucous coat of the appendix was found to be enormously hypertrophied, and the main mass of the tumour was made up of mucoid material, probably derived from the mucosa of the appendix, although the author does not attempt to explain how this mucus got into the peritoneal cavity. The case is to be classed among the rare pseudo-myxomata which are sometimes found intraperitoneally. The patient made an excellent recovery.

